# Chemical Synthesis and Biological Activities of Amaryllidaceae Alkaloid Norbelladine Derivatives and Precursors

**DOI:** 10.3390/molecules27175621

**Published:** 2022-08-31

**Authors:** Marie-Pierre Girard, Vahid Karimzadegan, Marianne Héneault, Francis Cloutier, Gervais Bérubé, Lionel Berthoux, Natacha Mérindol, Isabel Desgagné-Penix

**Affiliations:** 1 Département de Chimie, Biochimie et Physique, Université du Québec à Trois-Rivières, Trois-Rivières, QC G8Z 4M3, Canada; 2 Département de Biologie Médicale, Université du Québec à Trois-Rivières, Trois-Rivières, QC G8Z 4M3, Canada

**Keywords:** Amaryllidaceae alkaloid, norbelladine, dengue virus, anti-cholinesterase, specialized metabolism, *O*-methylation, galanthamine, Alzheimer’s disease, antiviral

## Abstract

Amaryllidaceae alkaloids (AAs) are a structurally diverse family of alkaloids recognized for their many therapeutic properties, such as antiviral, anti-cholinesterase, and anticancer properties. Norbelladine and its derivatives, whose biological properties are poorly studied, are key intermediates required for the biosynthesis of all ~650 reported AAs. To gain insight into their therapeutic potential, we synthesized a series of *O*-methylated norbelladine-type alkaloids and evaluated their cytotoxic effects on two types of cancer cell lines, their antiviral effects against the dengue virus (DENV) and the human immunodeficiency virus 1 (HIV-1), and their anti-Alzheimer’s disease (anti-cholinesterase and -prolyl oligopeptidase) properties. In monocytic leukemia cells, norcraugsodine was highly cytotoxic (CC_50_ = 27.0 μM), while norbelladine was the most cytotoxic to hepatocarcinoma cells (CC_50_ = 72.6 μM). HIV-1 infection was impaired only at cytotoxic concentrations of the compounds. The 3,4-dihydroxybenzaldehyde (selectivity index (SI) = 7.2), 3′,4′-*O*-dimethylnorbelladine (SI = 4.8), 4′-*O*-methylnorbelladine (SI > 4.9), 3′-*O*-methylnorbelladine (SI > 4.5), and norcraugsodine (SI = 3.2) reduced the number of DENV-infected cells with EC_50_ values ranging from 24.1 to 44.9 μM. The *O*-methylation of norcraugsodine abolished its anti-DENV potential. Norbelladine and its *O*-methylated forms also displayed butyrylcholinesterase-inhibition properties (IC_50_ values ranging from 26.1 to 91.6 μM). Altogether, the results provided hints of the structure–activity relationship of norbelladine-type alkaloids, which is important knowledge for the development of new inhibitors of DENV and butyrylcholinesterase.

## 1. Introduction

Amaryllidaceae are a family of monocotyledonous plants of the order Asparagales, which is composed of 1100 species and 75 genera that are found in tropical, subtropical, and warm regions of the world [1]. For centuries, Amaryllidaceae plant extracts have been recognized worldwide for their varied therapeutic properties, including anti-acetylcholinesterase (AChE), anti-microbial, and anti-tumor properties [2,3,4,5]. Their medicinal potency is mostly attributed to the presence of specialized metabolites of the alkaloid group, which are named the Amaryllidaceae alkaloids (AAs) [2,6]. To date, more than 650 AAs have been reported and classified into norbelladine-, cherylline-, galanthamine-, lycorine-, lycorenine-, crinine-, narciclasine-, tazettine-, and montanine-type based on the proposed biosynthetic origin of the ring structure and their carbon skeleton [1,6,7]. All AAs are derived from norbelladine, which is a common metabolic intermediate formed through the condensation of tyramine and 3,4-dihydroxybenzaldehyde (3,4-DHBA) [8,9].

**Figure 1:**
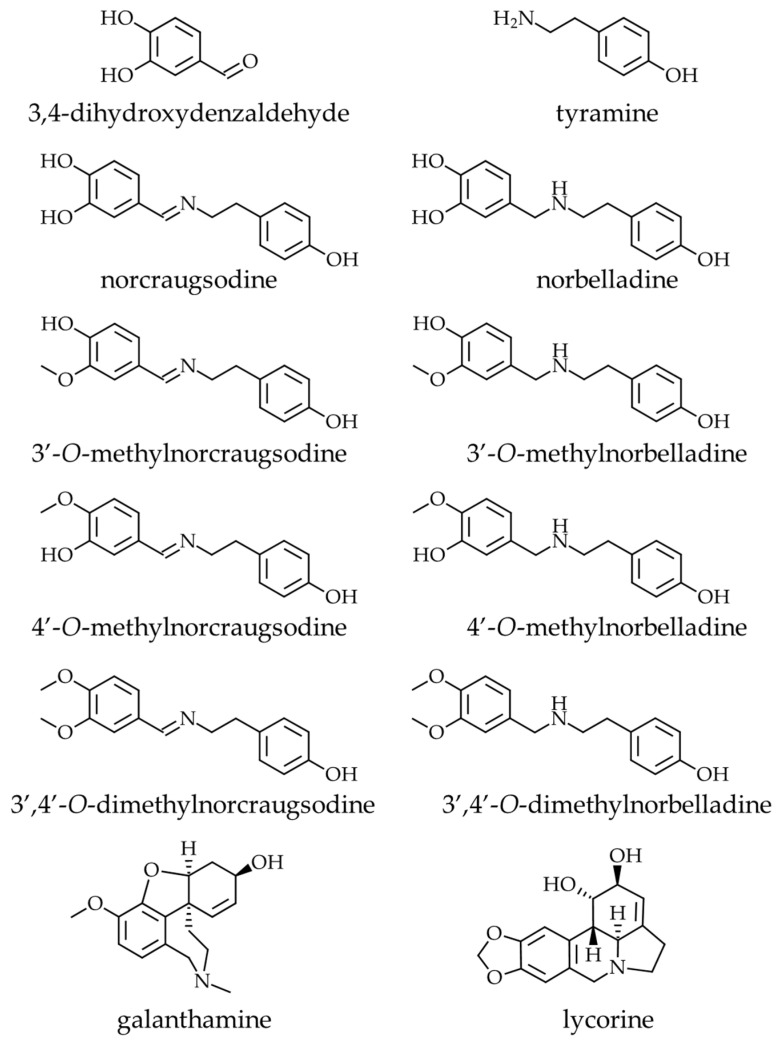
Chemical structures of the molecules used in this study. Amaryllidaceae alkaloid precursors (3,4-dihydroxybenzaldehyde and tyramine), intermediates (norcraugsodine and norbelladine), their corresponding *O*-methylated derivatives (3′-*O*-methylnorcraugsodine, 3′-*O*-methylnorbelladine, 4′-*O*-methylnorcraugsodine, 4′-*O*-methylnorbelladine, 3′,4-’*O*-dimethylnorcraugsodine, and 3′,4′-*O*-dimethylnorbelladine), and the well-known AAs galanthamine and lycorine.

AAs are known to have various pharmacological properties [10]. For example, several AAs, such as sanguinine and galanthamine (Figure 1), are strong anti-acetylcholinesterase inhibitors, with the latter being currently used as a treatment for symptoms of Alzheimer’s disease (AD) [11,12]. Lycorine, like many other AAs, exhibits anticancer (i.e., cytotoxic activity) [13,14,15,16,17], but also exerts an inhibitory effect against flaviviruses, such as DENV (dengue virus), and viruses belonging to other families [18,19,20,21]. Recently, we demonstrated that the AAs cherylline, pancracine, haemanthamine, and haemanthidine display antiviral effects against DENV, and except for cherylline, also against human immunodeficiency virus (HIV-1) [22,23]. The development of antiviral therapeutics based on AAs may provide decisive medical solutions to catastrophic pandemics. Since many of these molecules exert their antiviral action by targeting host factors, they present opportunities to develop broad-spectrum treatments that are less susceptible to the emergence of drug resistance [24].

To date, few studies have been performed on the biological potential of norbelladine-type AAs. One study demonstrated that norbelladine itself has slight in vitro anti-inflammatory and anti-oxidant properties [25]. In another study, synthetically designed complex alkaloid derivatives of carltonin A and B of the norbelladine-type were shown to exhibit anti-butyrylcholinesterase (BuChE) and -prolyl oligopeptidase (POP) properties, both of which are considered interesting targets for AD [26,27,28]. However, the pharmaceutical properties of norbelladine-type alkaloids in relation to AD, viral infections, and cytotoxicity remain largely unknown.

In this study, we investigated the biological activities of norbelladine-type molecules. Since Amaryllidaceae plants do not accumulate high levels of such metabolites, we report on the chemical synthesis of norbelladine, norcraugsodine, and their *O*-methylated derivatives (i.e., 3′-*O*-methylnorbelladine, 3′-*O*-methylnorcraugsodine, 4′-*O*-methylnorbelladine, 4′-*O*-methylnorcraugsodine, 3′,4′-*O*-dimethylnorbelladine, and 3′,4′-*O*-dimethylnorcraugsodine) (Figure 1). We assessed their antiviral potential in cellulo, as well as that of their precursors, namely, 3,4-dihydroxybenzaldehyde (3,4-DHBA) and tyramine, using propagative DENV_GFP_ vector and a non-propagative HIV-1_GFP_ vector. We analyzed their cytotoxicity against acute monocytic leukemia THP-1 cells and hepatocytic-cellular-carcinoma-derived Huh7 cells. We also measured their anti-AD potential through an assessment of anti-AChE, -BuChE, and -POP activity. We report for the first time that 3,4-DHBA and *O*-methylated norbelladine derivatives inhibited DENV infection and that norbelladine displayed anti-butyrylcholinesterase activity.

## 2. Results

### 2.1. Chemical Synthesis

The synthesis of norbelladine and the different methylated analogs was performed following a two-step reaction sequence reported in the literature for norbelladine [9,25]. Initially, the condensation of the relevant aldehyde with tyramine led to the imide intermediates with excellent yields (98% to 100%). Next, simple catalytic hydrogenation allowed us to generate the final derivatives with yields ranging from 43% to 99%. 

### 2.2. Cytotoxic Assay

**Figure 2:**
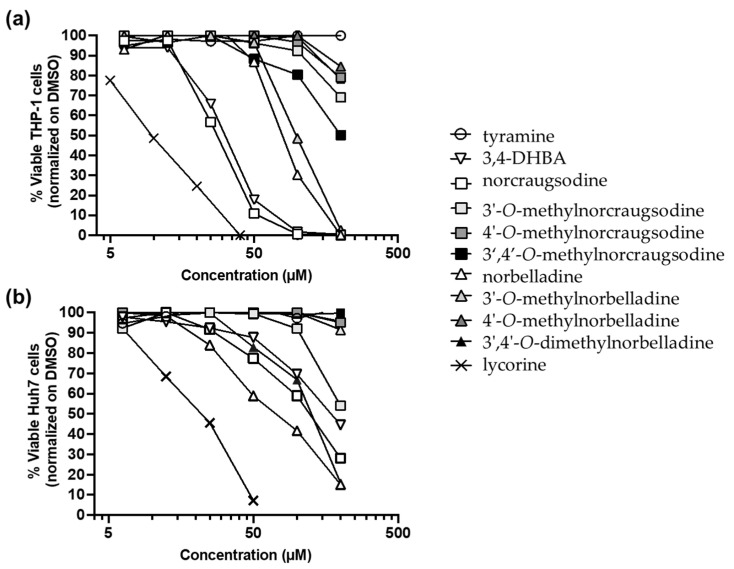
Cytotoxic effects of norbelladine precursors and their derivatives on Huh7 and THP-1 cells. To assess the cell viability, the cellular ATP levels were measured on (**a**) THP-1 and (**b**) Huh7 cells 72 h after alkaloid addition at concentrations of 6.25 µM to 200 µM. Lycorine was utilized as a positive control at concentrations of 0.3 µM to 40 µM. Results were normalized to equivalent concentrations of DMSO and the *x*-axis is displayed in log_10_. DMSO: dimethylsulfoxide; ATP: adenosine triphosphate.

Several alkaloids of the Amaryllidaceae family were reported to be cytotoxic [29]. Therefore, we evaluated the cytotoxic activity of the AA precursors, namely, 3,4-DHBA and tyramine, and the eight norbelladine-derived molecules on two types of cancer cell lines, including human monocytic leukemia cells (THP-1) and human hepatocarcinoma cells (Huh7). Tyramine is a natural by-product of the breakdown of the amino acid tyrosine and is found in plants and animals. As expected for both cell lines, tyramine was not cytotoxic and did not affect the cell viability, whereas lycorine was cytotoxic [30] at concentrations below 50 µM (Figure 2, Table 1). For THP-1 cells, norcraugsodine and 3,4-DHBA at 100 µM and norbelladine and 3′-*O*-methylnorbelladine at 200 µM were highly cytotoxic, killing a majority of the cells (Figure 2a). For Huh7 cells, norbelladine, norcraugsodine, 3′,4′-*O*-dimethylnorbelladine, and 3,4-DHBA at 200 µM were cytotoxic, producing 50% cell death (Figure 2b). 

The median cytotoxic concentrations (CC_50_) that caused a 50% decrease in cell viability were estimated for all alkaloids reaching this value (Figure 2, Table 1). Norcraugsodine, 3,4-DHBA, norbelladine, and 3′-*O*-methylnorbelladine were highly cytotoxic to THP-1 cells, with CC_50_ values ranging from 27.0 µM to 99.0 µM (Table 1). The 3′- and 4′-*O*-methylnorcraugsodine, 3′-*O*-methylnorbelladine, 3′,4′-*O*-dimethylnorcraugsodine, and 4′-*O*-methylnorbelladine exhibited only moderate cytotoxicity (<50% of cell death) at 200 µM on THP-1 cells (Figure 2a, Table 1). The norbelladine, norcraugsodine, 3′,4′-*O*-dimethylnorbelladine, and 3,4-DHBA were cytotoxic to Huh7 cells (Figure 2b), with CC_50_ values ranging from 72.6 µM to 173.1 µM (Table 1). The 3′-*O*-methylnorcraugsodine was weakly cytotoxic to Huh7 cells at the tested concentrations (Figure 2b). All other tested molecules were not cytotoxic (Figure 2b; Table 1). 

In summary, 3,4-DHBA, norcraugsodine, and norbelladine were the most cytotoxic to both types of cell lines, whereas 3′-*O*-methylnorbelladine displayed cytotoxic specificity to THP-1, and 3′,4′-*O*-dimethylnorbelladine to Huh7 cells (Figure 2; Table 1). 

### 2.3. Antiviral Assay

**Figure 3:**
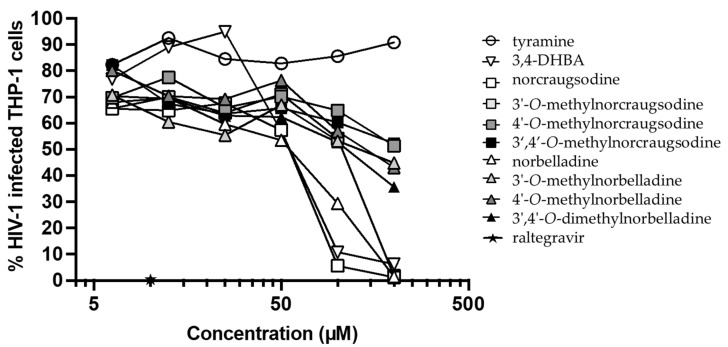
Antiretroviral effect of norbelladine precursors and derivatives on HIV-1_GFP_. The antiviral activities against HIV-1_GFP_ of norbelladine precursors and derivatives were evaluated 72 h post-infection using THP-1 cells via flow cytometry at concentrations ranging from 6.25 µM to 200 µM. Infections were performed with non-propagative HIV-1_GFP_ virus at a multiplicity of infection (MOI) of 0.1. Raltegravir served as a positive control and DMSO as a negative control at concentrations equivalent to the tested alkaloids. Results were normalized to the value with HIV-1_GFP_ infection without treatment and the *x*-axis is displayed as log_10_.

**Figure 4:**
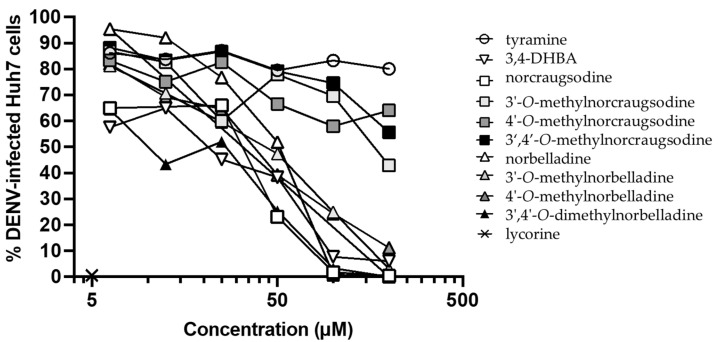
Antiflaviviral effects of norbelladine precursors and derivatives on DENV_GFP_. The antiviral activities against DENV_GFP_ of norbelladine precursors and derivatives were evaluated 72 h post-infection using Huh7 cells via flow cytometry at concentrations ranging from 6.25 µM to 200 µM. Infections were performed with propagative DENV_GFP_ virus at an MOI of 0.025. Lycorine was used as a positive control and DMSO as a negative control at concentrations equivalent to the tested alkaloids. Results were normalized to the value of DENV_GFP_ infection without treatment and the *x*-axis is displayed as log_10_.

**Figure A1:**
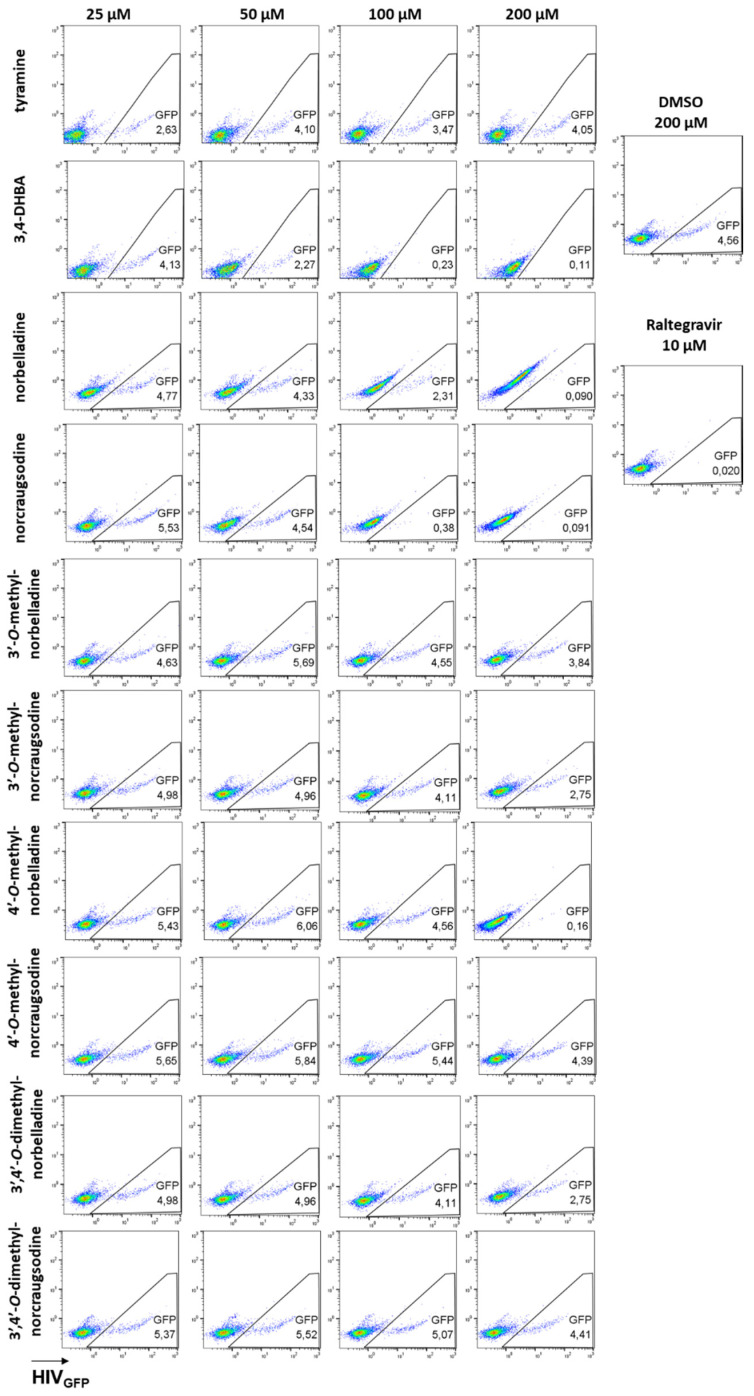
Antiretroviral effects of four concentrations (25, 50, 100, and 200 µM) of norbelladine precursors and derivatives on HIV-1_GFP_ in THP1 cells. Representative dot plots are presented. DMSO (200 µM) and raltegravir (10 µM) were included as the negative and positive controls, respectively.

**Figure A2:**
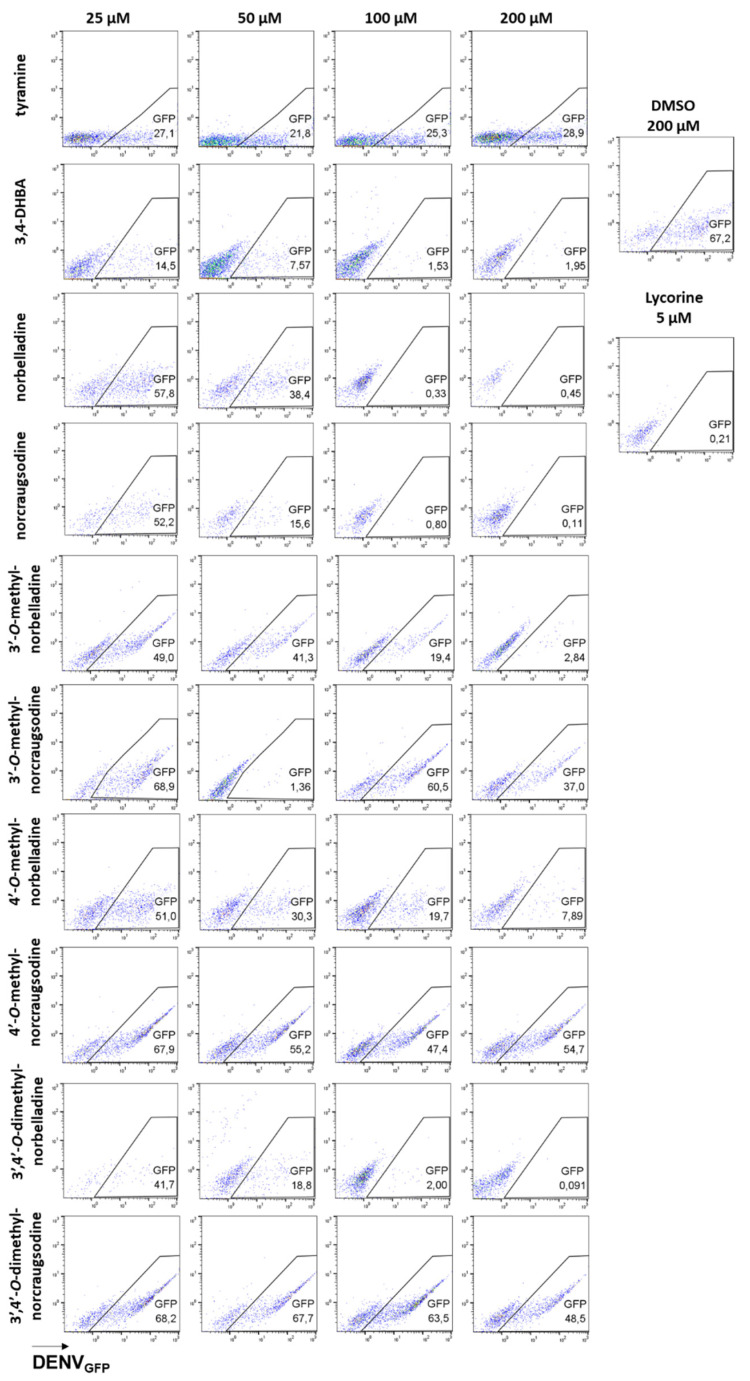
Antiflaviviral effects of norbelladine precursors and derivatives on DENV_GFP_. The antiviral activity against DENV_GFP_ was evaluated 72 h post-infection using Huh7 cells via flow cytometry at four concentrations (25, 50, 100, and 200 µM) of norbelladine precursors and derivatives. Lycorine (5 µM) was included as the positive control and DMSO (200 µM) as the negative control. Representative dot plots are presented.

Several studies shed light on the outstanding antiviral properties of alkaloids extracted from Amaryllidaceae, such as lycorine, cherylline, haemanthamine, haemanthidine, and pancracine [17,21,22]. Hence, we measured the antiviral activity of 3,4-DHBA, tyramine, and the eight norbelladine-derived molecules toward HIV-1_GFP_ and DENV_GFP_ in THP-1 and Huh7 cells, respectively. Infection levels were measured 72 h post-infection, where a dose-dependent inhibition of HIV-1_GFP_ (Figure 3, Appendix A Figure A1) and DENV_GFP_ (Figure 4, Appendix A Figure A2) was generated. In addition, the effective concentration inhibiting infection by 50% (EC_50_) was calculated, along with the selectivity index (SI) that was determined by the ratio of CC_50_ and EC_50_ (Table 1).

#### 2.3.1. Inhibition of HIV-1_GFP_

Norcraugsodine, 3,4-DHBA, and norbelladine prevented HIV-1 infection in most cells at 100 µM, while their methylated forms and tyramine were mostly inactive. At 200 μM, all three compounds and 3′-O-methylnorcraugsodine impeded HIV-1 infection (from 94% to 99% inhibition) (Figure 3, Appendix A Figure A1), with EC_50_ values ranging from 50.5 µM to 107.2 µM, and selectivity indices (SIs) ranging from 0.5 to 1.6 (Table 1). Hence, at these concentrations, AAs were also associated with significant toxicity, raising concerns about the specificity of their antiretroviral properties.

#### 2.3.2. Inhibition of DENV_GFP_

3,4-DHBA, 3′,4′-*O*-dimethylnorbelladine, norcraugsodine, 4′-*O*-methylnorbelladine, 3′-*O*-methylnorbelladine, and norbelladine showed strong inhibition of DENV_GFP_ infection at 50, 100, and 200 µM. Huh7 cells treated with 200 µM of these compounds resulted in an 89% to 100% decrease in infection, with the EC_50_ values ranging from 24.1 µM to 50.4 µM, and SI ranging from 1.5 to 6.2 (Table 1, Figure 4; Appendix A Figure A2). 3,4-DHBA, 4′-*O*-methylnorbelladine, 3′,4′-*O*-dimethylnorbelladine, and 3′-*O*-methylnorbelladine were the most selective with SI > 4.5. The other alkaloids tested showed little or no specific antiviral effect against DENV_GFP_ infections.

### 2.4. Choline Esterase and Prolyloligopeptidase Inhibitory Effect

**Figure 5:**
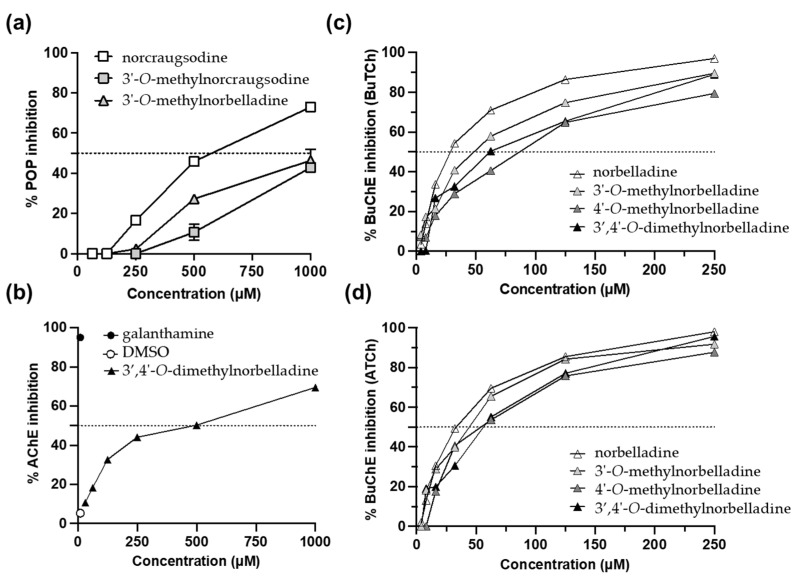
Anti-Alzheimer’s disease properties. (**a**) Prolyl oligopeptidase inhibition by norcraugsodine, 3′-O-methylnorcraugsodine, and 3′-O-methylnorbelladine. (**b**) Acetylcholinesterase inhibition of 3′,4′-O-methylnorbelladine using acetylthiocholine as the substrate. (**c**) Butyrylcholinesterase (BuChE) inhibition of norbelladine and derivatives using butyrylthiocholine (BuTCh) as the substrate. (**d**) Butyrylcholinesterase inhibition of norbelladine and derivatives using acetylthiocholine (ATCh) as the substrate. Galanthamine (10 μM) was used as the positive control for the AchE assays, while rivastigamine (2 mM) was used for the BuChE assays (100% inhibition not shown on the graph).

Enzymatic inhibitions were first trialed using 1 mM of compounds in duplicates (Appendix A Table A1). Further experiments were carried out only on selected active molecules. Of all the compounds tested, only norcraugsodine, 3′-*O*-methylnorcraugsodine, and 3′-*O*-methylnorbelladine inhibited POP activity, where norcraugsodine was the most potent with IC_50_ = 463.8 μM (Figure 5a). Among all the AAs and precursors, only 3′,4′-*O*-dimethylnorbelladine exhibited a moderate level of inhibition of AChE, with IC_50_ = 319.6 μM (Figure 5b). Among the 10 molecules tested, only norbelladine and its methylated forms significantly blocked BuChE activity using both acetylthiocholine and butyrylthiocholine as the substrate (Figure 5c,d). Norbelladine was the most potent, with IC_50_ values of 33.26 μM and 26.13 μM, respectively (Appendix A Table A1). The addition of two methyl groups in the 3′- and 4′-*O* positions lead to a ~3-fold decrease in inhibition.

### 2.5. Molecular Docking of Norbelladine Derivatives with BuChE

**Figure 6:**
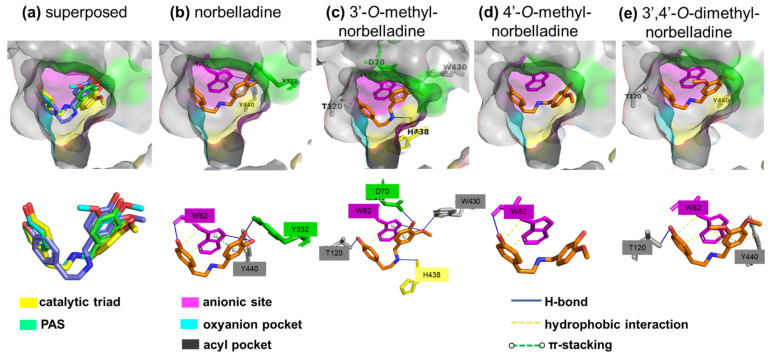
Prediction of norbelladine derivatives’ interactions with butyrylcholinesterase. Grey surface representation of BuChE (4BDS) active site with key subsites highlighted in different colors (catalytic triad is in yellow, pre-anionic site (PAS) is in green, anionic site is in pink, oxyanion pocket is in turquoise, and the acyl pocket is in dark grey). (**a**) Superimposition of docked ligands in the BuChE active site (norbelladine is in green, 3′-*O*-methylnorbelladine is in purple, 4′-*O*-methylnorbelladine is in yellow, and 3′,4′-*O*-dimethylnorbelladine is in turquoise). (**b**) Non-covalent (H-bond, hydrophobic, and P-stack) interactions of norbelladine with Trp82 from the anionic site, Tyr332 from the PAS, and Tyr440 from the binding site. (**c**) Non-covalent interactions of 3′-*O*-methylnorbelladine with W82 from the anionic site, Asp70 from the PAS, His438 from the catalytic triad, and Trp430 from the binding site. (**d**) Non-covalent interactions of 4′-*O-*methylnorbelladine with Trp82 from the anionic site. (**e**) Non-covalent interactions of 3′,4′-*O*-dimethylnorbelladine with Trp82 from the anionic site and Thr120 and Tyr440 from the binding site.

To better understand the interactions between the norbelladine derivatives and BuChE, we performed docking using the crystal structure of human BuChE (PDB: 4BDS) (Table 2, Figure 6). The active site of BuChE is located at the bottom of a profound gorge (20 Å) comprising six conserved aromatic residues and six aliphatic (Leu286 and Val288) and polar residues [28]. It includes a catalytic triad (Ser198, Glu325, and His438) that mediates the choline esters hydrolysis; an anionic site (Trp82, Tyr128, Phe329), which is essential for the reaction; and an oxyanion hole (Gly116, Gly117, Ala199) that stabilizes the transition state through hydrogen-bond interactions. Meanwhile, the acyl pocket (Ala199, Leu286, and Val288) is responsible for substrate specificity [31], and the peripheral anionic site (PAS or P-site) (Asp70, Tyr332) at the entry of the active gorge, which interacts with the cationic substrate guiding them down the gorge to the catalytic triad, is implicated in interactions with beta-amyloid and in the binding with many inhibitors [32,33] (Figure 6). The docking scores were very similar for norbelladine and its derivatives, ranging from −6.68 to −7.03 kCal/mol (Table 2). All the molecules interacted with the key residue of the anionic site Trp82. The hydrophobic and H-bonds that interacted with Trp82 were stabilized by aromatic π-π stacking in the case of norbelladine and 3′-O-methylnorbelladine, which is consistent with other inhibitors, such as tacrine [34], while the docking conformation of the 4′-*O*-methylated compounds did not allow for such an interaction. In the case of norbelladine and 3′-*O*-methylnorbelladine, the interaction with BuChE was also supported with H-bonds with PAS amino acids (Tyr332 and Asp70, respectively), and with other binding site residues (Tyr440 for norbelladine, Thr120 and Trp430 for 3′-*O*-methylnorbelladine). In addition, 3′-*O*-methylnorbelladine was H-bonded to the catalytic residue His438. Thus, the docking results were consistent with inhibition mechanisms like those of other previously reported inhibitor molecules, such as tacrine, and possibly reflected a stronger and more stable inhibition potential for norbelladine and 3′-*O*-methylnorbelladine compared with the 4′-O-methylated norbelladine derivatives (Figure 5 and Figure 6).

## 3. Discussion

Norbelladine and its *O*-methylated forms are mandatory intermediates in the biosynthesis of Amaryllidaceae alkaloids [8,35], yet their biological properties remain poorly studied. Norbelladine itself was shown to possess anti-inflammatory and cyclooxygenase inhibitory effects [25], while 3′-*O*-methylnorbelladine and a few complex synthetic derivatives of norbelladine and belladine were shown to display anti-cholinesterase activity [26].

We obtained the precursor imines norcraugsodine, 3′-*O*-methylnorcraugsodine, 4′-*O*-methylnorcraugsodine, and 3′,4′-*O*-dimethylnorcraugsodine, from which we made norbelladine [9], 3′-*O*-methylnorbelladine, 4′-*O*-methylnorbelladine, and 3′,4′-*O*-dimethylnorbelladine via catalytic hydrogenation. Their chemical synthesis was a straightforward process and the products were generally obtained in good to excellent yields.

Cytotoxic assays revealed that 3,4-DHBA, norcraugsodine, and norbelladine were the most cytotoxic compounds, both in monocytic leukemia THP-1 cells and hepatocarcinoma Huh7 cells (Figure 2, Table 1). Interestingly, the 3′-*O*-methylnorbelladine was selectively toxic to THP-1 cells, while 3′,4′-*O*-dimethylnorbelladine was selectively toxic to Huh7, hinting toward different mechanisms of cytotoxicity between the two cell lines.

Norbelladine, norcraugsodine, and 3,4-DHBA also strongly impeded HIV-1_GFP_ and DENV_GFP_ infections (Table 1, Figure 3 and Figure 4). The concentrations required to inhibit HIV-1 infection were cytotoxic to THP-1 cells (Table 1, Figure 3). Hence, the decrease in HIV-1 infection caused by these alkaloids was more consistent with progressive depletion of cell viability rather than a specific antiviral effect. In the case of DENV, viral inhibition was more specific, occurring at non-cytotoxic doses. Some non-cytotoxic alkaloids, such as 3′- and 4′-*O*-methylnorbelladine, also efficiently inhibited DENV_GFP_ replication at >100 µM. The 3′-*O*-methylnorcraugsodine, 4′-*O*-methylnorcraugsodine, and 3′,4′-*O*-dimethylnorcraugsodine showed little or no antiviral activity in contrast to norcraugsodine, revealing the importance of *O*-methylation to the toxic and antiviral nature of alkaloids (Table 1, Figure 4).

Several studies demonstrated the antiviral effect of AAs against several types of viruses [18,19,20,21,22,23,36,37,38]. Here, the virus used to perform DENV_GFP_ infections corresponded to dengue serotype 2. Recently, we also uncovered that the Amaryllidaceae alkaloids haemanthamine, pancracine, and haemanthidine, which were isolated from *Pancratium maritimum*, inhibited DENV-2 infection [23]. This study added to the growing evidence that AA structures could be optimized to develop potent inhibitors against this potentially fatal disease. Future studies should address the potency of these compounds toward other serotypes (DENV-1, -3, and -4), and other flaviviruses [39,40].

Investigation of the inhibitory properties of alkaloids on POP, AChE, and BuChE activity confirmed that the *O*-methylation and reduction state of AAs significantly affected their potencies. POP is a post-proline cleaving enzyme of the central nervous system, whose alteration is implicated in memory loss; Alzheimer’s, Parkinson’s, and Huntington’s diseases; and other neurodegenerative diseases [41]. Norcraugsodine, 3′-*O*-methylnorcraugsodine, and 3′-*O*-methylnorbelladine were the only compounds to inhibit POP at high concentrations >500 μM (Figure 5). Mamun et al. also reported limited efficiency of synthetic norbelladine and belladine derivatives to inhibit this enzyme [26].

AChE activity is dominant in regulating acetylcholine levels in healthy brains, while in an Alzheimer’s disease (AD) patient’s brain, the activity of BuChE is increased [42]. Thus, both enzymes are considered major therapeutic targets that can be used to fight AD. With the exception of 3′,4′-*O*-dimethylnorbelladine, none of the molecules inhibited AChE activity in our experiments. The use of DMSO as a solvent could have masked their activity up to a certain level. However, norbelladine and *O*-methylated derivatives consistently inhibited the BuChE-catalyzed hydrolysis of both butyrylcholine and acetylcholine. Norbelladine was the most potent, while there was a 3-fold loss of inhibition in the case of 3′,4′-*O*-dimethylnorbelladine. These results, which were in line with Mamun et al.’s screening [26], emphasized the interest in norbelladine as a backbone when developing butyrylcholinesterase inhibitors.

BuChE is a serine hydrolase enzyme that can hydrolyze several choline esters, including acetylcholine, succinylcholine, and butyrylcholine [28]. Although its role is not fully understood, its levels are increased in AD patients and it could promote amyloid plaque formation [43]. The active site of this enzyme consists of (1) the catalytic site composed of Ser198, His438, and Glu325 [44]; (2) an acyl pocket with Ala199, Leu286, and Val288 interacting with an acyl group of the esters [45]; (3) an anionic site with Trp82 that binds to a quaternary nitrogen of choline; and (4) an enzyme gorge lip consisting of Asp70 and Tyr332 guiding substrate toward the catalytic site [46].

In a study conducted by Nachon et al. (2013), human BuChE was crystalized in a complex with tacrine, which is a strong inhibitor of BuChE. According to this study, aromatic π-π stacking between tacrine and Trp82 is essential for its inhibitory effect [47]. A similar interaction was predicted by docking norbelladine and its *O*-methylated forms with BuChE, which was consistent with the relatively strong inhibitory effect observed in our in vitro enzymatic assays (Figure 6). Furthermore, an additional interaction between Tyr332 and the hydroxyl group of norbelladine ring was obtained in our model that can potentially reduce the accessibility of BuChE to the substrate since Tyr332 was proposed to direct the substrate to the active site of the enzyme. It should be noted that a weak interaction between Tyr332 and tacrine was also observed [47].

## 4. Materials and Methods

### 4.1. Chemical Synthesis and Purification of Alkaloids 

The starting material, reactant, and solvents were obtained commercially and used as such or purified and dried using standard methods [48]. The infrared spectra were recorded on a Nicolet Impact 420 FT-IR spectrophotometer. Nuclear magnetic resonance (NMR) spectra were recorded on a Varian 200 MHz NMR apparatus. Samples were dissolved in dimethyl sulfoxide (DMSO)-d6 for data acquisition using the residual solvent signal as an internal standard (*δ* 2.49 ppm for ^1^H NMR and 39.95 ppm for ^13^C NMR). Chemical shifts (δ) are expressed in parts per million (ppm), whereas the coupling constants (J) are expressed in hertz (Hz). Multiplicities are described using the following abbreviations: s for singlet, d for doublet, t for triplet, m for multiplet, and bs for broad singlet.

Two-step synthesis of norbelladine and methylated analogs:

Norcraugsodine, norbelladine, 3′-*O*-methylnorcraugsodine, 3′-*O*-methylnorbelladine, 4′-*O*-methylnorcraugsodine, 4′-*O*-methylnorbelladine, 3′,4′-*O*-dimethylnorcraugsodine, and 3′,4′-*O*-dimethylnorbelladine were obtained using organic synthesis following a two-step reaction sequence as described below. The products were characterized via infrared (IR) spectroscopy, as well as proton (^1^H NMR) and carbon nuclear magnetic resonance (^13^C NMR) spectroscopy.

**Step 1: General procedure for the preparation of the imine intermediates.**

An equimolar quantity of the relevant benzaldehyde and tyramine were added as powders to a flask containing dichloromethane (20 mL). The solution was stirred gently overnight (about 12 h) at room temperature to yield the imine intermediate. The solvent was evaporated under reduced pressure using a rotatory evaporator, followed by mechanical pumping to remove the residual solvent and water. The resulting imines were obtained in excellent yields and were sufficiently pure to be used as such in the hydrogenation step.

***Norcraugsodine*:** Step 1 with 3,4-dihydroxybenzaldehyde (537 mg, 3.88 mmol), tyramine (533 mg, 3.88 mmol), and dichloromethane (20 mL). Norcraugsodine (0.99 g, 99%). IR (cm^−1^): 3345 (OH), 3038 (aromatic), and 1648 (C=N); ^1^H NMR (200 MHz, DMSO-d6) *δ*: 7.98 (1H, s, CH imine), 7.15 (1H, d, *J* = 2.0 Hz, CH-Ar), 6.99 (2H, d, *J* = 8.6 Hz, CH-Ar), 6.90 (1H, dd, J_1_ = 2 Hz and J_2_ = 8.2 Hz, CH-Ar), 6.65 (1H, d, *J* = 8.6 Hz, CH-Ar), 6.63 (2H, d, *J* = 8.6 Hz, CH-Ar), 3.63 (2H, t, *J* = 7.2 Hz, CH=NCH_2_CH_2_), 2.73 (2H, t, *J* = 7.4 Hz, CH=NCH_2_CH_2_); ^13^C NMR (200 MHz, DMSO-d6) *δ*: 160.9, 155.9, 149.5, 146.1, 130.5, 130.1, 127.8, 121.9, 115.8, 115.4, 113.9, 62.4, and 36.8.

***3′-O-methylnorcraugsodine*:** Step 1 with 4-hydroxy-3-methoxybenzaldehyde (vanillin) (561 mg, 3.68 mmol), tyramine (506 mg, 3.68 mmol), and dichloromethane (20 mL). 3′-*O*-Methylnorcraugsodine (0.98 g, 98%). IR (cm^−1^): 3008 (OH) and 1639 (C=N); ^1^H NMR (200 MHz, DMSO-d6) *δ*: 8.37 (1H, s, CH imine), 6.99 (4H, m, CH-Ar), 6.69 (3H, m, CH-Ar), 3.73 (2H, m, CH=NCH_2_CH_2_ and 3H, s, OMe), 2.79 (2H, t, *J* = 7.03 Hz, CH=NCH_2_CH_2_); ^13^C NMR (200 MHz, DMSO-d6) *δ*: 166.4, 156.1, 152.6, 148.6, 130.1, 129.7, 123.5, 118.5, 117.8, 115.5, 114.9, 59.9, 56.1, and 36.3.

***4′-O-methylnorcraugsodine*:** Step 1 with 3-hydroxy-4-methoxybenzaldehyde (isovanillin) (561 mg, 3.68 mmol), tyramine (506 mg, 3.68 mmol), and dichloromethane (20 mL). 4′-*O*-methylnorcraugsodine (1.00 g, 100%). IR (cm^−1^): 3508, 2900 (OH), and 1638 (C=N); ^1^H NMR (200 MHz, DMSO-d6) *δ*: 8.8 (1H, OH), 8.04 (1H, s, CH imine), 7.19 (1H, d, *J* = 1.56 Hz, CH-Ar), 6.97 (4H, m, CH=Ar), 6.65 (2H, d, *J* = 8.2 Hz, CH-Ar), 3.77 (3H, s, OMe), 3.64 (2H, t, *J* = 7.03Hz, CH=NCH_2_CH_2_), and 2.74 (2H, t, *J* = 7.03 Hz, CH=NCH_2_CH_2_); ^13^C NMR (200 MHz, DMSO-d6) *δ*: 160.8, 155.9, 150.4, 147.1, 130.5, 130.1, 129.8, 121.2, 115.4, 113.6, 111.9, 62.8, 55.9, and 36.7.

***3′,4′-O-dimethylnorcraugsodine*:** Step 1 with 3,4-dimethoxybenzaldehyde (582 mg, 3.50 mmol), tyramine (481 mg, 3.50 mmol), and dichloromethane (20 mL). 3′,4′-*O*-dimethylnorcraugsodine (1.00 g, 100%); IR (cm^−1^): 2938 (OH) and 1638 (C=N); ^1^H NMR (200 MHz, DMSO-d6) *δ*: 9.05 (1H, OH), 8.13 (1H, s, CH imine), 7.32 (1H, s, CH-Ar), 7.15 (1H, d, *J* = 7.81 Hz, CH-Ar), 6.99 (3H, m, CH-Ar), 6.66 (2H, d, *J* = 8.2 Hz, 3.77 (6H, s, 2 x OMe), 3.67 (2H, t, *J* = 7.03 Hz, CH=NCH_2_CH_2_), and 2.77 (2H, t, *J* = 7.03 Hz, CH=NCH_2_CH_2_); ^13^C NMR (200 MHz, DMSO-d6) *δ*: 160.7, 155.9, 151.4, 149.4, 130.4, 130.1, 129.6, 122.8, 115.4, 111.6, 109.4, 62.9, 55.9, 55.8, and 36.7.

**Step 2: General procedure for the preparation of the final amine products.**

The relevant imine was dissolved in a mixture of ethylacetate/methanol (9:1, 10 mL) and hydrogenated to the amine using 30 mol% palladium on carbon (Pd/C 10%) under a H_2_ atmosphere using a balloon. The hydrogen was bubbled three times (t = 0, 30, and 60 min) during the hydrogenation process. The mixture was agitated for 2 to 3 h (or until the disappearance of the starting material via TLC) and then filtered on a silica gel using ethylacetate/methanol (4:1 mixture) to remove the Pd/C. The solvent was evaporated under reduced pressure using a rotatory evaporator, followed by mechanical pumping to yield the desired amine. The final amines were obtained in yields ranging from 43% to 98%.

***Norbelladine*:** Step 2 with norcraugsodine (150 mg, 0.58 mmol), under a H_2_ atmosphere, 10% Pd/C (40 mg), and ethyl acetate/methanol (10 mL). Norbelladine (130 mg, 86%). IR (cm^−1^): 3021 (-OH and NH); ^1^H NMR (200 MHz, DMSO-d6) *δ*: 6.94 (2 H, d, *J* = 8.6 Hz, CH-Ar), 6.63 (4 H, m, CH-Ar), 6.52 (1H, dd, J_1_ = 1.7 Hz and J_2_ = 7.7 Hz, CH-Ar), 3.49 (2H, s, Ar-CH_2_-NH), and 2.58 (4 H, m, NH-CH_2_CH_2_-Ar); ^13^C NMR (200 MHz, DMSO-d_6_) *δ*: 155.8, 145.4, 144.3, 132.1, 130.9, 129.8, 119.2, 116.0, 115.6, 115.5, 53.1, 51.1, and 35.4.

***3′-O-methylnorbelladine*:** Step 2 with 3′-*O*-methylnorcraugsodine (182.5 mg, 0.67 mmol), under H_2_ atmosphere, 10% Pd/C (40 mg), and ethyl acetate/methanol (10 mL). 3′-O-methylnorbelladine (182.5 mg, 99%). IR (cm^−1^): 2934 (OH and NH); ^1^H NMR (200 MHz, DMSO-d6) *δ*: 6.98–6.65 (7H, m, CH-Ar), 3.81 (2H, s, Ar-CH_2_-NH), 3.71 (3H, s, OMe), and 2.64 (4H, m, NH-CH_2_CH_2_-Ar); ^13^C NMR (200 MHz, DMSO-d6) *δ*: 160.8, 152.7, 151.8, 134.8, 134.6, 129.1, 126.0, 123.2, 120.3, 116.4, 60.8, 55.2, 55.1, and 39.4.

***4′-O-methylnorbelladine*:** Step 2 with 4′-*O*-methylnorcraugsodine (266 mg,), under H_2_ atmosphere, 10% Pd/C (40 mg), and ethyl acetate/methanol (10 mL). 4′-O-methylnorbelladine (114 mg, 43%). IR (cm^−1^): 2989 (OH and NH); ^1^H NMR (200 MHz, DMSO-d6) *δ*: 6.96–6.62 (7H, m, CH-Ar), 3.71 (3H, s, OMe), 3.55 (2H, s, Ar-CH_2_-NH), and 2.60 (4H, br s, NH-CH_2_CH_2_-Ar); ^13^C NMR (200 MHz, DMSO-d6) *δ*: 155.9, 146.8, 146.7, 133.6, 130.7, 129.8, 119.0, 115.9, 115.5, 112.4, 56.1, 52.9, 51.0, and 35.3.

***3′,4′-O-dimethylnorbelladine*:** Step 2 with 3′,4′-*O*-dimethylnorcraugsodine (404 mg, 1.41), under H_2_ atmosphere, 10% Pd/C (70 mg), and ethyl acetate/methanol (10 mL). 3′,4′-*O*-dimethylnorbelladine (400 mg, 98%). IR (cm^−1^): 3261 (OH) and 2953 (OH and NH)); ^1^H NMR (200 MHz, DMSO-d6) *δ*: 6.97–6.55 (7H, m, CH-Ar), 3.69 (6H, s, OMe), 3.60 (2H, br s, Ar-CH_2_-NH), and 2.61 (4H, br s, NH-CH_2_CH_2_-Ar); ^13^C NMR (200 MHz, DMSO-d6) *δ*: 155.9, 149.0, 147.9, 133.9, 130.9, 129.8, 120.2, 115.4, 112.1, 111.9, 55.9, 55.8, 53.0, 51.1, and 35.4.

### 4.2. Preparation of Lycorine, 3,4-DHBA, Tyramine, and other Commercial Inhibitor Stocks

Lycorine was isolated from *Crinum jagus* according to the method reported in [49] and was provided by Antonio Evidente (Universita di Napoli Federico II, Naples, Italy). 3,4-DHBA (purity: 97%, formula: C_7_H_6_O_3_, molar mass: 138.12, CAS number: 139-85-5) was obtained from Acros Organics. Tyramine (purity: ≥98%, formula: C_8_H_11_NO, molar mass: 137.18, CAS number: 51-67-2) was obtained from Sigma Aldrich. Raltegravir (purity: 99.85%, formula: C_20_H_20_FN_6_O_5_K, molar mass: 482.52, CAS number: 871038-72-1) was obtained through the NIH HIV Reagent Program, Division of AIDS, NIAID (Isentress; MK-0518). Each compound was dissolved in dimethylsulfoxide (DMSO) at a final concentration of 100 mM and stored at −20 °C until subsequent use. Rivastigmine and galanthamine hydrobromide were purchased from Millipore Sigma (Sigma-Aldrich, Oakville, ON, Canada) and solubilized in water at 20 mM just before the experiments.

### 4.3. Cell Lines and Culture

The human hepatocarcinoma Huh7 cell line was kindly provided by Hugo Soudeyns (University of Montréal, Montréal, QC, Canada). Huh7, Crandell-Rees Feline Kidney Cell (CRFK), and Vero cells were maintained in Dulbecco’s Modified Eagle Medium (DMEM) supplemented with 10% fetal bovine serum (FBS) and 1% penicillin/streptomycin solution (all from Wisent, Inc., Saint-Jean-Baptiste, QC, Canada). The human leukemia monocytic THP-1 cell line was maintained in Roswell Park Memorial Institute (RPMI) medium supplemented with 10% fetal bovine serum (FBS) and 1% penicillin-streptomycin solution (all from Wisent, Inc., Saint-Jean-Baptiste, QC, Canada). All cell lines were kept in an incubator at 37 °C and 5% CO_2_.

### 4.4. Cytotoxicity Assay

Cytotoxicity assays of norbelladine and its derivatives were performed on Huh7 and THP-1 cells by measuring ATP levels using the Cell-Titer GLO assay kit (Promega, Madison, WI, USA). Briefly, 7.5 × 10^3^ Huh7 cells/well or 2 × 10^4^ THP-1 cells/well were plated in black 96-well plates and incubated at 37 °C for 24 h. The next day, precursors, norbelladine, its derivatives, DMSO, and lycorine were serially diluted by a factor of 2 in DMEM complete medium (for Huh7) or RPMI complete medium (for THP-1) at room temperature. Each dilution was added to the cell plates to obtain final concentrations of 6.25 µM to 200 µM for alkaloids and DMSO, and 0.39 µM to 50 µM for lycorine. DMSO was used as a negative control, while lycorine was used as a positive control since its cytotoxic effect was previously demonstrated [50]. The plates were then incubated at 37 °C and 5% CO_2_ for 72 h. Cell-Titer GLO reagent was added to the plates previously equilibrated to room temperature. The plates were then mixed on an orbital shaker for 2 min and incubated for 10 min at room temperature. The luminescence signal was measured with a microplate spectrophotometer (Synergy H1, Biotek, Dorval, QC, Canada). Viability percentages were obtained by calculating the ratio of the signal corresponding to each alkaloid concentration to the signal of the equimolar DMSO control. All experiments were performed at least twice. Median cytotoxic concentrations (CC_50_) were calculated using QuestGraph IC50 calculator software (MLA Quest Graph™ IC_50_ Calculator, AAT Bioquest, Inc., Sunnyvale, CA, USA).

### 4.5. Viral Vectors

To investigate the antiviral effect of norbelladine and its derivatives, we used a dengue virus propagative vector (DENV_GFP_) and a non-propagative human immunodeficiency virus (HIV)-1 pseudotyped VSV-G vector (HIV-1_GFP_), both of which encoded green fluorescent protein (GFP). The plasmid used to obtain the DENV_GFP_ vector (pFK-DVs-G2A) was provided by Ralf Bartenschlager (Heidelberg University, Heidelberg, Germany) and Laurent Chatel-Chaix (Institut National de la Recherche Scientifique, Laval, QC, Canada) [22,51]. The 2 plasmids used to obtain the HIV-1_GFP_ vector were PMD2.G and pNL4-3-GFPΔEnvΔNef [52]. For DENV_GFP_, viral titer was measured using a plaque assay in Vero cells, as described in [53]. For HIV-1_GFP_, the viral titer was obtained by measuring the infectivity of serially diluted vectors in CRFK cells, as described in [54].

### 4.6. Antiviral Assays 

Briefly, 7.5 × 10^3^ Huh7 cells/well or 2 × 10^4^ THP-1 cells/well were plated in 96-well plates at 37 °C for 24 h. The next day, norbelladine and its derivatives, as well as the DMSO-dissolved lycorine (Huh7) or DMSO-dissolved raltegravir (THP-1), were serially diluted by a factor of 2 in DMEM or RPMI medium, respectively. Each dilution was added to the cell plates to obtain final concentrations of 1.56 µM to 200 µM for alkaloids and matched concentrations of DMSO, and 0.05 µM to 6.4 µM for lycorine or 0.078 µM to 10 µM for raltegravir. Lycorine and raltegravir were used as DENV and HIV-1 inhibitor controls, respectively [18,55]. DENV_GFP_ and HIV-1_GFP_ were then added at multiplicities of infection (MOIs) of 0.025 and 0.1, respectively. Plates were placed in an incubator at 37 °C and 5% CO_2_ for 72 h. Afterward, the cells were fixed in 3.7% formaldehyde and the percentage of infection was measured using flow cytometry with an FC500 MPL cytometer (Beckman Coulter, Inc., Brea, CA, USA). Data analysis was performed using Flowjo software (BD, FlowJo LLC, Ashland, OR, USA). All experiments were performed at least twice. EC_50_ values were calculated using QuestGraph IC50 calculator software (MLA Quest Graph™ IC50 Calculator. AAT Bioquest, Inc. https://www.aatbio.com/tools/ic50-calculator (accessed on 1 July 2022)).

### 4.7. Anti-acetylcholinesterase (AChE) and -Butyrylcholinesterase (BuChE) Activity

Pharmacological properties specific to AD were tested first on AChEs (electric eel) according to the kit (ab138871, Abcam) that provides a colorimetric measure of enzyme activity and inhibition. Briefly, the reaction was performed in a final volume of 100 μL in 96-well microplates. A preliminary screen to identify the most potent AChE inhibitors was performed. For this, DMSO-dissolved test compounds were added to a final concentration of 1 mM (1% DMSO) in duplicates. Next, a 5 μL reaction mixture containing equal amounts of acetylthiocholine (20X) and DTNB (20X) was added to each well. Ultimately, the enzyme solution was added to a final concentration of 0.25 U/mL and the absorbance was measured at 412 nm in kinetic mode for 10 min using a microplate reader (Synergy H1, Biotek, Dorval, QC, Canada). The same procedure was utilized for the BuChE (equine, Sigma-Aldrich) activity test, except that the enzyme concentration was 2 U/mL per reaction. DTNB (Bis(3-carboxy-4-nitrophenyl) disulfide, Ellman’s Reagent), acetylthiocholine iodide, and butyrylthiocholine iodide were purchased from Sigma-Aldrich (Oakville, ON, Canada). Galanthamine (10 μM) and rivastigamine (2 mM) were used as positive controls for the AChE and BuChE assays, respectively. Molecules showing inhibition during preliminary screenings were selected for further assessment of IC_50_ values using serially diluted concentrations. Experiments were performed at least twice. Inhibition was calculated as follows [26]:

I = 100 × (1 − Δi/Δe)

where Δi is the difference in the absorbance between two time points in the presence of the inhibitor, and Δe is the difference in the absorbance using two time points in presence of DMSO or an appropriate solvent.

### 4.8. Prolyloligopeptidase (POP) Inhibition Assay

POP enzyme activity was measured using the Fluorogenic POP Assay Kit (BPS Bioscience Inc., San Diego, CA, USA). The reaction was carried out in a final volume of 50 μL in a low-binding NUNC microtiter plate. All compounds were tested at a final concentration of 250 μM in duplicate. A DPP substrate was added to a final concentration of 2.5 μM and the POP enzyme solution was subsequently added to a final concentration of 1 ng/μL. Optical densities at 440 nm were recorded using a microplate reader (Synergy H1, Biotek, QC, Canada).

### 4.9. Docking of Norbelladine Derivatives with BuChE

Docking was performed using the crystal structure of human BuChE in a complex with tacrine (PDB: 4BDS) using MOE 2020.09 software (Chemical Computing Group). Tacrine was removed. Structure issues were corrected using the structure preparation tool and amino acids were protonated using the protonate3D tool. Ligands were protonated at pH = 7 using the protomer option. Ligands were protonated at pH = 7 using the protomers tool. The active site (Asn68, Ile69, Asp70, Gln71, Ser72, Gly78, Ser79, Trp82, Tyr114, Gly115, Gly116, Gly117, Gln119, Thr120, Gly121, Thr122, Leu125, Tyr128, Glu197, Ser198, Ala199, Trp231, Glu276, Ala277, Val280, Gly283, Thr284, Pro285, Leu286, Ser287, Val288, Asn289, Phe290, Ala328, Phe329, Tyr332, Phe398, Trp430, Met437, His438, Gly439, Tyr440, Ile442) was predicted using the site finder tool of the MOE software and validated with literature data. Dummy atoms across the active site were created and used as docking sites. Water and solvent molecules were removed, residues further than 8 Å from dummy atoms were fixed, and active site residues were tethered using the QuickPrep default parameters. The triangle matcher method was used to place ligands in the active site using the London dG score with 200 poses, and an induced fit was used as a refinement option with 10 poses and the GBVI/WSA score. The first pose of the most abundant configuration was chosen to be conserved for the ligand–protein interaction analysis. This corresponded to the 1st pose for all ligands except 3′,4′-*O*-methylnorbelladine, for which the 1st pose displayed a flipped structure; hence, the 2nd pose was selected. The Protein–Ligand Interaction Profiler (PLIP) was used to analyze the interaction of the ligands with the binding site following the docking procedure [56]. Pymol (Schrödinger) was used to visualize and present the PLIP results.

### 4.10. Statistical Analysis

All the analyses and related graphs were performed and produced, respectively, using GraphPad Prism version 8.0.0 (GraphPad Software, San Diego, CA, USA).

## 5. Conclusions

In summary, precursors and norbelladine derivatives did not exhibit antiviral effects against HIV-1_GFP_ infections. However, they did possess appreciable inhibitory activity against DENV_GFP_ infections and butyrylcholinesterase.

The antiviral and inhibitory activities detected in this study were detected at doses associated with some cytotoxicity. These compounds should be optimized to increase their selectivity index. In future studies, viral targets should be identified to ascertain the mechanism of the compounds’ antiviral activity. This would enable structure–activity relationship analysis, which is necessary knowledge for designing optimal antiviral drugs and preventing the development of a drug-resistant virus.

Some of the compounds could still have activity against HIV-1 that we would not have detected, as the pseudotyped particles used in this study did not include HIV-1 entry and release steps. In addition, the anti-flavivirus activity should be confirmed using wild-type dengue and Zika viruses. Finally, considering the broad activity of Amaryllidaceae alkaloid in general, other RNA viruses will be tested in future studies.

The results obtained in this study increased our knowledge of the structure–activity relationship of alkaloids with a norbelladine backbone. They provide further insight into the biological potency of norbelladine-type molecules, depending on the nature and location of the different *O*-methyl groups. This new knowledge could help to better guide the selection and optimization of AAs for the development of new inhibitors to fight Alzheimer’s disease and infections caused by flaviviruses.

## Figures and Tables

**Table 1:** EC_50_, CC_50_, and SI values of norbelladine precursors and derivatives with antiviral effects. EC_50_: median effector concentration; CC_50_: median cytotoxic concentration; SI: selectivity index.

Alkaloids	EC_50_ HIV-1 (µM)	CC_50_ THP-1 (µM)	SI HIV-1	EC_50_ DENV (µM)	CC_50_ Huh7 (µM)	SI DENV
Tyramine	>200 ^-^	>200 ^-^	<1.0	>200 ^-^	>200 ^-^	<1.0
3,4-DHBA	51.5 *	31.65	0.6	24.1 *	173.1	7.2
Norbelladine	50.5	82.2	1.6	50.4	72.6	1.4
Norcraugsodine	55.5	27.0	0.5	37.7	121.8	3.2
3′-*O*-methylnorcraugsodine	107.2 *	>200	>1.9	176.3 *	>200	>1.1
3′-*O*-methylnorbelladine	134.7 *	99.01	0.73	44.9 *	>200	>4.5
4′-*O*-methylnorbelladine	108.3 *	>200	>1.8	40.5 *	>200	>4.9
4′-*O*-methylnorcraugsodine	>200 ^-^	>200 ^-^	<1.0	>200 ^-^	>200 ^-^	<1.0
3′,4′-*O*-dimethylnorbelladine	98.3 *	>200	>2.0	27.5	131.4	4.8
3′,4′-*O*-dimethylnorcraugsodine	>200 ^-^	>200 ^-^	<1.0	>200 ^-^	>200 ^-^	<1.0
Raltegravir	0.098	>10 ^-^	>102	u.i.	u.i.	u.i.
Lycorine	u.i.	10.7	u.i.	0.090	17.4	193.3

EC_50_ and CC_50_ values of antiviral compounds (norbelladine, norcraugsodine, 3′-*O*-methylnorcraugsodine, 3′-*O*-methylnorbelladine, 4′-*O*-methylnorbelladine, 3′,4′-*O*-dimethylnorbelladine, and 3,4-DHBA) were calculated using the QuestGraph IC50 calculator (MLA Quest Graph™ IC50 Calculator, AAT Bioquest, Inc.). SI = CC_50_/EC_50_. “-”: EC_50_ or CC_50_ was not achieved. “*”: compound addition did not yield complete viral inhibition. “u.i.”: unidentified.

**Table 2:** Predictions of norbelladine derivatives’ interactions with butyrylcholinesterase.

	Score (kCal/mol)	Interaction		
		H-bonds	Hydrophobic	π stack
Norbelladine	−6.6829	Trp82, Tyr332, Tyr440	Trp82, Tyr440	Trp82
3′-*O*-Methylnorbelladine	−6.8174	Asp70, Trp82, Thr120, Trp430, His438	Trp82	Trp82
4′-*O*-Methylnorbelladine	−6.8119	Trp82	Trp82	n.d.
3′,4′-*O*-Dimethylnorbelladine	−7.0368	Thr120	Trp82, Tyr440	n.d.

n.d.: none detected.

## Data Availability

The data presented in this study are available on request from the corresponding author.

## Sample Availability

Samples of the compounds are not available from the authors.

## Appendix A

**Table A1:** Inhibitory properties (IC_50_ values) of norbelladine precursors and derivatives toward enzymes implicated in Alzheimer’s disease.

	POP	AChE	BuChE	
	DPP	ATCh	BuTCh	ATCh
**3,4-DHBA**	nid	nid	nid	nid
**Tyramine**	nd	nid	nid	nid
**Norcraugsodine**	567.30	nid	nid	nid
**Norbelladine**	nid	nid	26.13	33.26
**3′-*O*-Methylnorcraugsodine**	1146.01	nid	nid	nid
**4′-O-Methylnorcraugsodine**	nid	nid	nid	nid
**3′,4′-*O*-Dimethylnorcraugsodine**	nid	nid	nid	nid
**3′-*O*-Methylnorbelladine**	1230.60	nid	58.95	40.22
**4′-*O*-Methylnorbelladine**	nid	nid	91.61	42.92
**3′,4′-*O*-Dimethylnorbelladine**	nid	319.6	87.86	87.80

IC_50_ values are expressed in μM; nid stands for no inhibition detected at 1 mM. POP: prolyl oligopeptidase, DPP: Ala-Pro-AMC dipeptide, AChE: acetylcholinesterase, ATCh: acetylthiocholine, BuTCh: butyrylthiocholine, BuChE: butyrylcholinesterase.
